# Treatment Strategy for Subaxial Minimal Facet/Lateral Mass Fractures: A Comprehensive Clinical Review

**DOI:** 10.3390/jcm14082554

**Published:** 2025-04-08

**Authors:** Chae-Gwan Kong, Jong-Beom Park

**Affiliations:** Department of Orthopaedic Surgery, Uijeongbu St. Mary’s Hospital, The Catholic University of Korea College of Medicine, Uijeongbu 11765, Republic of Korea; gongjae@catholic.ac.kr

**Keywords:** treatment strategy, subaxial, facet/lamina, minimal fractures, vertebral artery injury

## Abstract

Minimal facet and lateral mass fractures of the subaxial cervical spine (C3–C7) are a distinct subset of spinal injuries that present diagnostic and therapeutic challenges. These fractures often result from low-energy trauma or hyperextension mechanisms. They are frequently stable. However, subtle fracture instability and associated soft tissue injuries may lead to delayed instability, neurological compromise, and/or chronic severe pain if not properly identified. Accurate diagnosis relies on a combination of plain radiography, high-resolution computed tomography (CT), and magnetic resonance imaging (MRI) to assess bony and ligamentous integrity. Treatment strategy is determined based on fracture stability, neurological status, and radiographic findings. Most stable fractures can be effectively treated with conservative treatment, allowing for natural healing while minimizing complications. However, when instability is suspected—such as those with significant disc and ligamentous injuries, progressive deformity, or neurological deficits—surgical stabilization may be considered. The presence of vertebral artery injury (VAI) can further complicate management. To mitigate the risk of stroke, a multidisciplinary approach that includes neurosurgery, vascular surgery, and interventional radiology is needed. Surgical treatment aims to restore spinal alignment, maintain stability, and prevent further neurological deterioration with approaches tailored to individual fracture patterns and patient-specific factors. Advances in surgical techniques, perioperative management, and endovascular interventions for VAI continue refining treatment options to improve clinical outcomes while minimizing complications. Despite increasing knowledge of these fractures and associated vascular injuries, optimal treatment strategies remain unclear due to limited high-quality evidence. This review provides a comprehensive analysis of the anatomy, biomechanics, classification, imaging modalities, and treatment strategies for minimal facet and lateral mass fractures in the subaxial cervical spine, highlighting recent advancements in diagnostic tools, therapeutic approaches, and managing vertebral artery injuries. A more precise understanding of the natural history and optimal management of these injuries will help spine specialists refine clinical decision-making and improve patient outcomes.

## 1. Introduction

Minimal facet and lateral mass fractures of the subaxial cervical spine (C3–C7) are commonly encountered in clinical practice. They represent a unique challenge in spinal trauma management. These fractures are often caused by low-energy traumas such as falls from standing height, minor motor vehicle accidents, and sporting injuries, with hyperextension and axial loading being predominant mechanisms. Recent studies have suggested that proper stability depends on several factors, including fracture morphology, integrity of surrounding ligamentous structures, and the presence of associated injuries such as traumatic disc herniation and ligamentous disruption [1,2,3,4,5,6,7,8]. However, they are traditionally considered stable injuries. Additionally, vertebral artery injury (VAI) is increasingly recognized as a potential complication of these fractures, with incidence rates ranging from 17% to 46%, particularly in cases involving facet fractures, subluxations, and dislocations [9,10,11,12,13,14,15,16].

The management of minimal facet and lateral mass fractures of the subaxial cervical spine remains an area of clinical controversy and evolving understanding. While these injuries are often considered stable and managed conservatively, recent studies have raised concerns about subtle instability associated with ligamentous disruption or traumatic disc herniation. Conflicting evidence exists regarding the optimal treatment for fractures classified as F1 and F2 injuries, with some advocating for early surgical intervention in cases of potential instability. The AO Spine Subaxial Facet Injury Classification provides a valuable framework for characterizing injury severity but lacks clear guidelines for managing intermediate or borderline cases, contributing to inconsistencies in treatment recommendations and outcomes reported in the literature [17,18,19].

**Figure 1:**
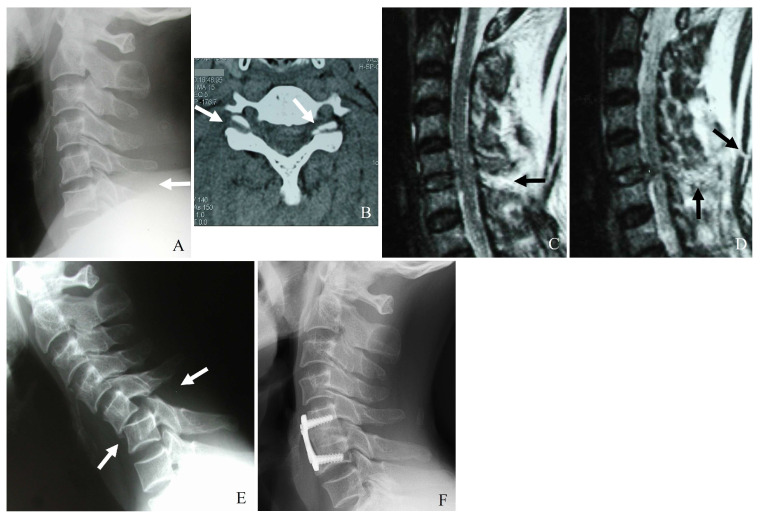
Plain X-ray (**A**), computed tomography (**B**), and magnetic resonance imaging (**C**,**D**) reveal a widening of the C5–C6 interspinous distance, a minimal fracture of the left facet, widening of the right facet joint (white arrows), traumatic herniation, and rupture of the posterior ligamentous complex at C5–C6 (dark arrows). Delayed bilateral facet dislocation of C5–C6 occurred 4 months after wearing a rigid brace (white arrows) (**E**). The patient underwent anterior cervical discectomy, open reduction, and fusion of C5–C6 (**F**).

Additionally, vertebral artery injury (VAI) is increasingly recognized as a significant concern in the context of subaxial cervical spine fractures. Reported incidence rates range from 17% to 46%, particularly in fractures involving facet dislocations, subluxations, or injuries extending to the transverse foramen. Given the potential for devastating complications such as ischemic stroke [20,21,22,23], the recognition and management of VAI are critical components of a comprehensive treatment approach (Figure 1) [20,21,22]. However, there remains a lack of consensus on the appropriate use of antithrombotic therapy, endovascular interventions, and radiological follow-up in the setting of VAI [24,25].

This review aims to synthesize current knowledge regarding the classification, imaging, and management of minimal facet and lateral mass fractures of the subaxial cervical spine. By addressing gaps in knowledge, evaluating conflicting evidence, and providing recommendations for both conservative and surgical management, this review seeks to optimize clinical decision-making and improve patient outcomes. Special attention is given to identifying and managing vertebral artery injury, which remains an underrecognized but critical factor influencing prognosis.

Given the limited number of high-quality prospective studies focused explicitly on the minimal facet and lateral mass fractures of the subaxial cervical spine, this review was designed as a narrative synthesis. We incorporated evidence from various sources, including retrospective studies, expert opinion, and case series, to reflect current clinical practice and highlight areas where further research is needed. While preparing this manuscript/study, the authors used ChatGPT-4o for English editing, reference text searching and preparation, and figure preparation. The authors have reviewed and edited the output and take full responsibility for the content of this publication.

## 2. Anatomy and Biomechanics of the Subaxial Cervical Spine

The subaxial cervical spine (C3–C7) provides stability and mobility, facilitating a balance between motion and protection of neural structures. This balance is achieved through the complex interplay of osseous structures, intervertebral discs, ligamentous components, and surrounding musculature. Facet joints and lateral masses play crucial roles in spinal stability. Facet joints, which articulate with adjacent vertebrae, are supported by the facet capsules, interspinous ligaments, and anterior and posterior longitudinal ligaments (ALL and PLL). The lateral mass serves as structural support, transmitting axial loads from the occiput and upper cervical spine to the lower vertebral column. Biomechanically, facet and lateral mass fractures occur due to axial loading, hyperextension, or rotational forces. Understanding the biomechanical implications of these fractures is crucial for determining treatment strategies and predicting outcomes [1,2,3,4].

### 2.1. Facet Joint Anatomy and Stability

Facet joints are synovial articulations between adjacent vertebrae’s superior and inferior articular processes. These joints are oriented at approximately 45 degrees in the coronal plane, facilitating controlled flexion, extension, and rotational movements while preventing excessive anterior translation. Each facet joint is enclosed by a fibrous capsule and supported by the ligamentum flavum, interspinous ligaments, and the PLL, collectively contributing to stability. The integrity of the facet joint is critical for maintaining the segmental motion of the cervical spine. Disruption of the facet capsule due to trauma or degenerative changes can result in increased motion and instability. Unilateral facet fractures often allow limited translation. However, they can progress to instability if associated with ligamentous injury. Bilateral facet fractures, on the other hand, are frequently unstable. They might result in significant subluxation or dislocation (Table 1) [5,6,7,8].

### 2.2. Disc and Ligamentous Complex in Stability

The intervertebral disc and ligamentous complex play a fundamental role in spinal stability. The anterior column consists of the ALL and vertebral bodies with intervertebral discs. The middle column includes the PLL and the posterior aspect of vertebral bodies, while the posterior column comprises facet joints, ligamentum flavum, and interspinous and supraspinous ligaments [5,6,7,8].

**Figure 2:**
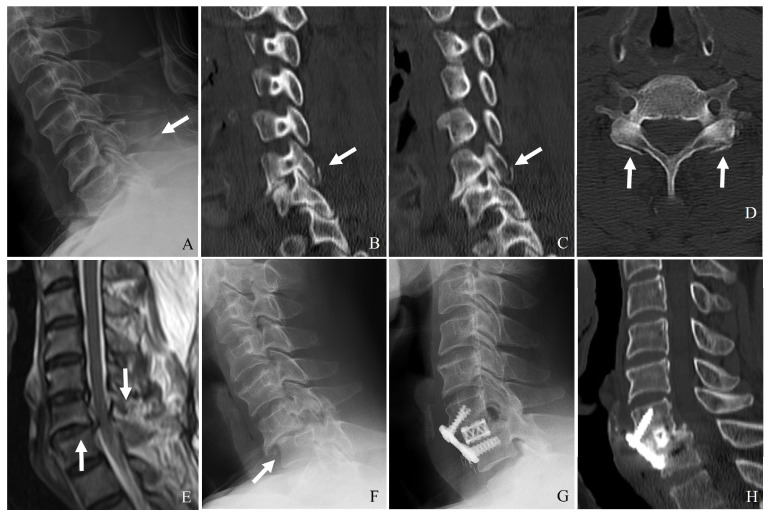
Plain X-ray (**A**), computed tomography (**B**–**D**), and magnetic resonance aging (**E**) demonstrate the widening of the interspinous distance, bilateral facet fractures, and injuries to the posterior ligamentous complex and posterior longitudinal ligament at C6–C7 (white arrows). Delayed bilateral facet dislocation of C6–C7 occurred 1 month after wearing a rigid brace (white arrows) (**E**,**F**). The patient underwent anterior cervical discectomy, open reduction, and fusion of C6–C7 (**G**,**H**).

ALL and PLL can prevent excessive extension and flexion, respectively, while the ligamentum flavum and interspinous ligaments provide posterior tension and prevent excessive separation of adjacent vertebrae. Injury to these structures, particularly in hyperextension or with hyperflexion mechanisms, can compromise stability and predispose patients to delayed deformity (Table 2) (Figure 2).

### 2.3. Mechanisms of Facet and Lateral Mass Fractures

Facet and lateral mass fractures primarily occur due to axial loading, hyperextension, or rotational forces. Axial loading can transmit force directly through the vertebral column, often resulting in compressive fractures of lateral masses. Hyperextension injuries can lead to avulsion fractures at the facet joint due to excessive tensile forces on anterior and posterior ligamentous structures. Rotational mechanisms, commonly seen in side impact injuries, can cause asymmetric facet disruption, leading to instability [1,2,3,4,5,6,7,8].

Understanding the biomechanical consequences of these injury mechanisms is essential for predicting long-term outcomes and guiding appropriate management. Although stable fractures could be managed conservatively, those associated with significant ligamentous disruption or facet subluxation often require surgical intervention.

## 3. Imaging Modalities

Accurate diagnosis of minimal facet and lateral mass fractures relies on high-resolution imaging techniques to assess both bony and ligamentous integrity. Imaging is pivotal in identifying fractures, determining stability, and guiding treatment decisions. Plain radiography, CT, and MRI are the primary imaging modalities for evaluating subaxial cervical spine injuries [1,2,3,4,5,6,7,8].

### 3.1. Plain Radiography

**Figure 3:**
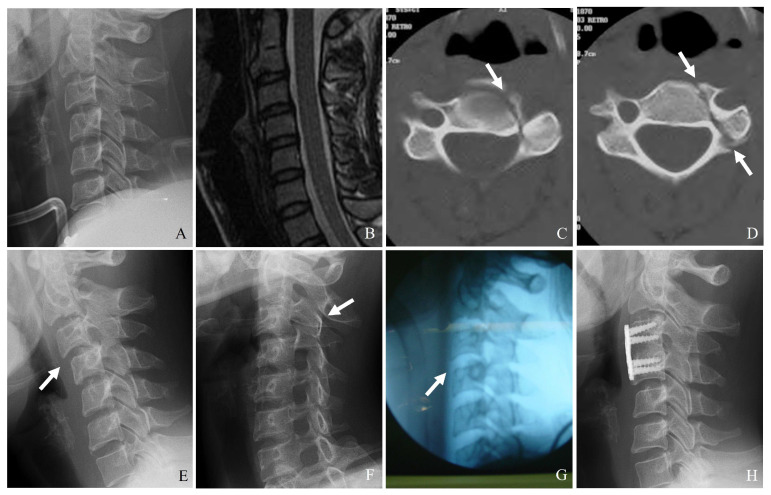
Plain X-ray (**A**) and magnetic resonance imaging (**B**) show no definite abnormalities. However, computed tomography (**C**,**D**) reveals a left facet fracture and fractures of the pedicle and body of C4 (white arrows). Delayed unilateral facet subluxation of C3–C4 occurred 1 month after wearing a rigid brace (white arrows) (**E**,**F**). The patient underwent closed reduction (white arrow) anterior cervical discectomy and fusion of C3–C4 (**G**,**H**).

Plain radiography serves as the initial imaging modality in cases of suspected cervical spine trauma. Standard anteroposterior and lateral views generally assess alignment and gross fractures. However, the sensitivity of radiographs in detecting subtle fractures, facet disruptions, and ligamentous injuries is limited, particularly in patients with degenerative changes or poor visualization due to overlying structures (Figure 3). Key radiographic signs that might indicate facet or lateral mass fractures include the following:

(1) Malalignment of posterior elements in the lateral view.
(2) Widening of the facet joint space, suggesting ligamentous disruption.
(3) Perched or dislocated facets may indicate instability requiring surgical intervention.
(4) Loss of vertebral height or step-off deformities, suggesting compression injuries.

Despite these advantages, plain radiographs are low-sensitivity for detecting subtle facet fractures. In addition, they are often insufficient for fully characterizing complex injuries. Consequently, advanced imaging is typically required for definitive assessment.

### 3.2. Computed Tomography (CT)

CT is the gold standard for diagnosing bony fractures in the cervical spine due to its high spatial resolution and ability to provide detailed cross-sectional imaging. Multidetector CT with thin-section reconstructions can enhance the visualization of subtle fractures, allowing for a more precise assessment of comminution, displacement, and stability (Figure 3) [21]. CT imaging is beneficial for:

(1) Identifying minimally displaced facet fractures that may be missed on radiographs.
(2) Assessing lateral mass comminution and fracture propagation.
(3) Evaluating facet joint alignment in axial and sagittal reconstructions.
(4) Determining the extent of impaction or subluxation.

### 3.3. Magnetic Resonance Imaging (MRI)

**Figure 4:**
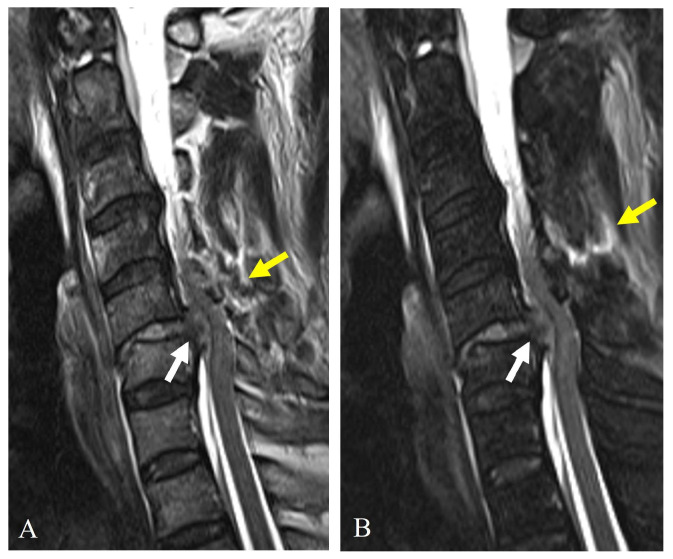
Magnetic resonance imaging (**A**,**B**) reveals traumatic disc rupture (white arrows) compressing the spinal cord and posterior ligament complex injury (yellow arrows) at C5–C6.

MRI is indispensable for evaluating soft tissue injuries, including ligamentous disruptions, disc herniation, and spinal cord involvement. Since the stability of facet fractures depends on the disc-ligamentous complex’s (DLC’s) integrity, MRI provides critical information that cannot be obtained from CT alone [22]. MRI is beneficial for (1) detecting injuries to the posterior ligamentous complex, which are crucial in determining instability; (2) evaluating traumatic disc herniations, which may necessitate surgical intervention; and (3) assessing spinal cord compression and potential myelopathy (Figure 4).

## 4. AO Spine Subaxial Facet Injury Classification

The AO Spine Subaxial Facet Injury Classification is a subcomponent of the AO Spine Subaxial Injury Classification System. It is specifically designed to categorize facet joint injuries in the C3–C7 region. This classification system provides a structured approach for assessing the severity of facet injuries and determining the appropriate management strategy. Facet injuries are categorized into four primary types (F1, F2, F3, and F4), reflecting the degree of disruption, instability, and displacement of the facet joint. The classification is crucial for differentiating between stable injuries that may be managed conservatively and unstable injuries that require surgical intervention [17,18].

### 4.1. Importance of Classification in Clinical Decision-Making

This structured classification system can aid in determining when surgical intervention is necessary, particularly in differentiating stable injuries (F1), moderately unstable injuries (F2), unstable fractures (F3), and comminuted unstable fractures (F4). It helps distinguish between stable injuries (F1), moderately unstable injuries (F2), and significantly unstable injuries (F3). Differentiation between F2 and F3 on imaging is crucial for treatment planning. F3 injuries almost always require surgical intervention, whereas F2 injuries may be managed conservatively depending on clinical factors.

Understanding radiological differences between F2 and F3 injuries in the AO Spine Subaxial Facet Injury Classification is essential for making informed treatment decisions and ensuring optimal patient outcomes. Although CT can identify gross displacement, MRI is essential for assessing ligamentous integrity and determining whether surgical intervention is warranted. By applying this structured classification, spine specialists can optimize patient outcomes, reduce unnecessary surgical interventions in stable injuries, and ensure early surgical stabilization in unstable cases (Table 3) [24,25].

### 4.2. Challenges in Differentiating Facet Fracture Types (F2 and F3)

The AO Spine Subaxial Facet Injury Classification System differentiates between displaced fractures without instability (F2) and displaced fractures with instability (F3). F2 and F3 fractures are distinguished based on fracture involvement and height. However, challenges arise due to the following issues [26,27,28]:

(1) Subjectivity in determining displacement and instability: The definition of “displacement” varies among studies, leading to inconsistencies in classification. Some authors argue that even F2 fractures (displaced but stable) may require fixation based on clinical judgment rather than classification.
(2) Lack of biomechanical validation: Limited biomechanical evidence distinguishes F2 from F3 fractures, making the classification somewhat reliant on subjective assessment rather than objective mechanical properties.
(3) Neurological considerations: The classification accounts for radiculopathy (N2), but in real practice, surgical decision-making is influenced by pain severity, duration, and response to conservative management—factors that the classification system does not fully capture.

### 4.3. Proposed Modifications and Future Directions

Given the concerns over reliability, several potential modifications have been suggested:

(1) Refinement of F2 vs. F3 criteria; a stricter, CT-based definition of facet displacement and instability could improve interobserver reliability.
(2) Integration of dynamic imaging: some authors propose incorporating extended X-rays or dynamic CT scans to better assess stability.
(3) Clinical modifiers: adding clinical parameters such as pain severity, response to conservative treatment, and facet capsule integrity could enhance decision-making.
(4) Machine learning-based classification, AI-driven models can analyze large datasets of facet injuries, which could improve the objectivity and reliability of classification in future research.

### 4.4. Interobserver Reliability and Limitations of Classification Systems

The AO Spine Subaxial Facet Injury Classification provides a structured framework for categorizing injuries based on fracture morphology, ligamentous disruption, and clinical instability. However, its application in clinical practice presents certain limitations.

#### 4.4.1. Interobserver Reliability

Studies assessing the interobserver reliability of the AO Spine Subaxial Facet Injury Classification have reported moderate to substantial agreement among spine specialists. However, variability remains, particularly in distinguishing between F2 (potentially unstable) and F3 (unstable) injuries. The subjective assessment of displacement, instability, and ligamentous injury can lead to inconsistent classification, mainly relying solely on CT imaging without supplementary MRI.

#### 4.4.2. Limitations in Clinical Practice

The classification system primarily relies on imaging findings, which may not fully capture the extent of ligamentous damage or dynamic instability. Discrepancies in fracture grading can affect clinical decision-making, particularly in borderline cases where surgical intervention may or may not be indicated. Limited biomechanical validation of the classification criteria further complicates its application in guiding treatment decisions. Integrating dynamic imaging techniques, such as flexion-extension X-rays or dynamic MRI, has been suggested to enhance diagnostic accuracy and interobserver agreement.

## 5. Conservative Treatment

**Figure 5:**
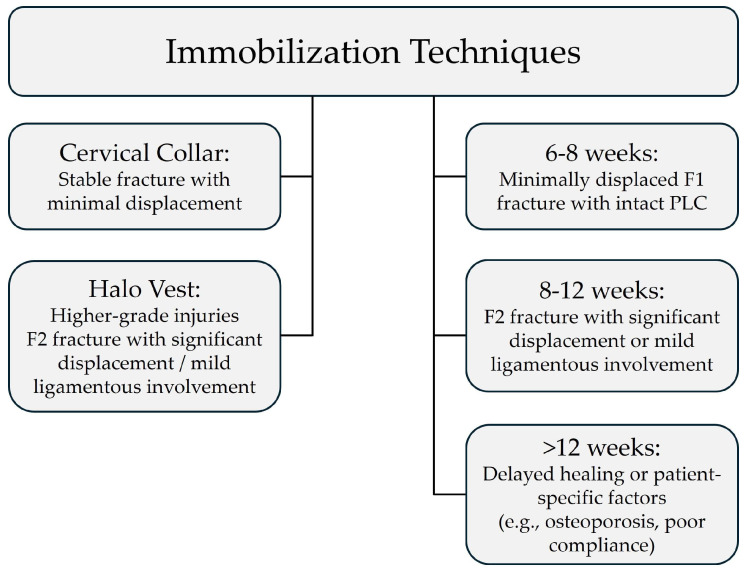
Flowchart explaining the immobilization techniques and duration based on the AO Spine Subaxial Facet Injury Classification.

Most stable facet and lateral mass fractures can be managed conservatively with cervical collar immobilization or a short-term orthosis, provided no significant displacement, ligamentous injury, or neurological impairment exists. The goal of conservative treatment is to allow for natural healing while minimizing complications such as post-traumatic kyphosis and chronic instability. When performing conservative treatment, immobilization techniques, duration of immobilization, physical therapy, and patient-specific factors influencing recovery are key considerations (Figure 5) [29,30,31].

### 5.1. Immobilization Techniques

Immobilization is essential to prevent further displacement and facilitate fracture healing. The choice of immobilization depends on the severity of the fracture, patient compliance, and associated injuries.

(1) Cervical collar: A rigid cervical collar is preferred for stable fractures with minimal displacement. It is suitable for unilateral, nondisplaced, or minimally displaced fractures without significant ligamentous injury (i.e., intact posterior ligamentous complex—PLC). It is worn for 6–8 weeks, with periodic clinical and radiographic evaluations.
(2) Halo vest immobilization: A halo vest is considered for higher-grade injuries where a higher level of immobilization is required (e.g., F2 injuries with significant displacement or mild ligamentous involvement). It is typically used for 8–12 weeks, followed by gradual weaning into a collar. It is not recommended for F3 injuries involving gross instability or complete PLC disruption because surgical intervention is generally required.

### 5.2. Duration of Immobilization

The length of immobilization varies depending on fracture healing, patient symptoms, and radiographic findings:

(1) Six to eight weeks: For minimally displaced F1 fractures with no ligamentous injury (intact PLC). Conservative management with a rigid cervical collar is usually sufficient.
(2) Eight to twelve weeks: For F2 fractures with significant displacement or mild ligamentous involvement, halo vest immobilization is typically applied for 8–12 weeks, with a gradual transition to a cervical collar if stability is achieved. However, if PLC disruption is extensive, surgical intervention may be required.
(3) Longer immobilization (>12 weeks): Considered in cases of delayed healing, patient-specific factors (e.g., osteoporosis, poor compliance), or where there is concern for potential instability despite conservative management.

Serial radiographic follow-up every 2–4 weeks is necessary to assess fracture healing and detect any delayed instability. If progressive displacement or worsening symptoms occur, a transition to surgical management may be required.

### 5.3. Patient-Specific Factors Influencing Conservative Treatment

Certain patient-related factors can influence the decision to pursue conservative management:

(1) Age and bone quality: Older patients with osteoporosis may have delayed healing and require prolonged immobilization.
(2) Compliance: Some patients may struggle with prolonged collar use, requiring adjustments in treatment strategy.
(3) Occupation and lifestyle: Athletes or individuals with physically demanding jobs may require more extended rehabilitation or early consideration of surgery.
(4) Associated injuries: If additional cervical trauma or soft tissue injury is present, a more conservative approach with extended monitoring is warranted.

### 5.4. Follow-Up and Transition to Surgery

Not all patients managed conservatively will heal without complications. Indications for surgical conversion include the following [29,30,31]:

(1) Progressive deformity or kyphosis on serial imaging.
(2) Persistent severe pain despite adequate immobilization.
(3) Delayed union or nonunion of the fracture.
(4) Neurological deterioration or worsening instability.

By carefully selecting patients for conservative management and ensuring following strict protocols, spine specialists can optimize healing while minimizing the risk of complications. The combination of immobilization, rehabilitation, and patient education remains the cornerstone of nonoperative treatment for stable facet and lateral mass fractures.

## 6. Surgical Treatment

Surgical intervention is indicated for unstable facet and lateral mass fractures, cases with neurological compromise, and failure of conservative treatment due to progressive deformity or persistent pain. Goals of surgical management include spinal realignment, stabilization, and decompression of neural elements when necessary. Treatment strategies depend on the instability severity, displacement degree, and associated ligamentous or disc injuries [32,33,34,35,36].

### 6.1. Indications for Surgery

Surgical stabilization is recommended in the following scenarios:

(1) Significant instability: Facet fractures classified as F3 or F4 in the AO Spine Subaxial Facet Injury Classification.
(2) Neurological deficit: Compression of the spinal cord or nerve roots, leading to weakness, sensory deficits, or radiculopathy.
(3) Progressive deformity: Increasing kyphotic angulation or worsening alignment on follow-up imaging.
(4) Failure of conservative management: Persistent severe pain, progressive subluxation, or delayed instability on serial imaging.

### 6.2. Surgical Approaches

Surgical strategies for facet and lateral mass fractures typically involve posterior fixation, anterior reconstruction, or a combination. The selection depends on fracture morphology, associated disc injury, and degree of instability.

#### 6.2.1. Posterior Fixation

Posterior fixation is the most commonly performed surgical approach for facet and lateral mass fractures due to its ability to restore spinal stability while preserving motion segments when possible. Standard techniques include the following:

(1) Lateral mass screws: This is preferred in subaxial cervical spine fixation due to their strong biomechanical support. They can provide rigid internal fixation while allowing controlled motion. They are used in isolated lateral mass fractures or unstable facet fractures.
(2) Pedicle screws: They offer stronger fixation than lateral mass screws, particularly in cases with severe comminution or vertebral instability. They are utilized when additional biomechanical stability is needed, such as in F4 fractures with severe facet fragmentation.
(3) Rod constructs and posterior fusion: They are used in bilateral facet dislocations or extensive ligamentous injury cases. Posterior fusion is indicated when long-term stability is required due to extensive ligamentous damage.

#### 6.2.2. Anterior Reconstruction

**Figure 6:**
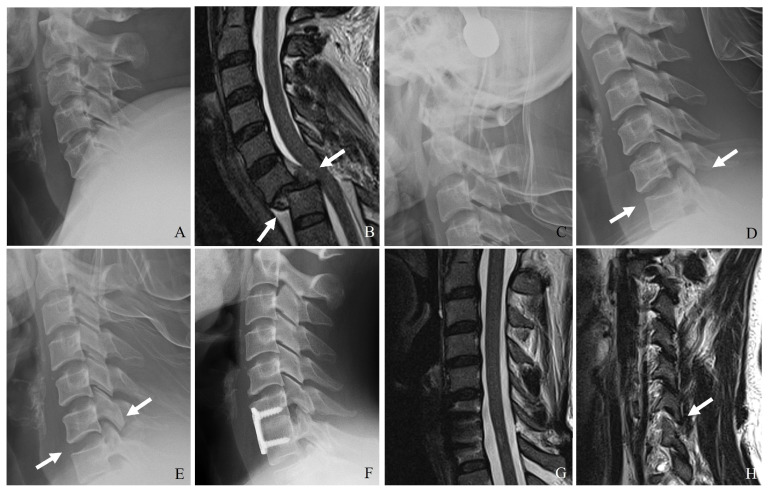
Plain X-ray (**A**) and magnetic resonance imaging (**B**) demonstrate bilateral facet dislocation and traumatic disc herniation at C6–C7 (white arrows). Successful closed reduction with skull traction was achieved (white arrows) (**C**–**E**). The patient subsequently underwent anterior cervical discectomy, open reduction, and fusion of C6–C7 (white arrow) (**F**–**H**).

Anterior surgical approaches are less commonly used. However, they might be necessary for cases involving significant disc injury or anterior column instability (Figure 6).

(1) Anterior cervical discectomy and fusion (ACDF): ACDF is indicated when facet fractures are associated with disc herniation or anterior subluxation. It can restore anterior column stability and decompress neural elements.
(2) Anterior cervical corpectomy and fusion (ACCF): ACCF is reserved for severe vertebral body comminution or multi-level instability. It requires structural grafting or cage placement to maintain spinal height.

#### 6.2.3. Combined Anterior-Posterior Stabilization

**Figure 7:**
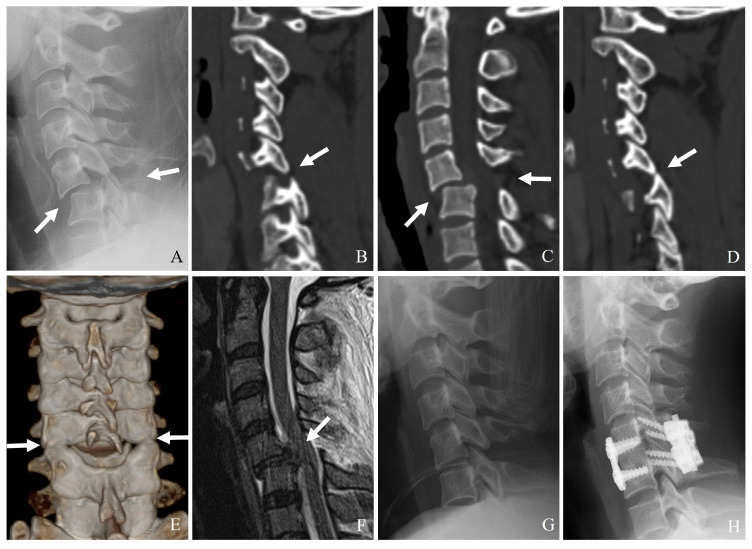
Plain X-ray (**A**), computed tomography (**B**–**E**), and magnetic resonance imaging (**F**) reveal bilateral facet dislocation and traumatic disc herniation at C5–6 (white arrows). Successful closed reduction with skull traction was achieved (**G**). The patient underwent combined anterior cervical discectomy, fusion, and posterior fixation of C5–C6 (**H**).

A combined anterior-posterior approach may be required for complex cases with multi-level instability or severe displacement (Figure 7).

(1) Anterior decompression and fusion (ACDF or ACCF) followed by posterior fixation provides superior stability.
(2) Indicated for severe F3 or F4 fractures with compromised ligamentous integrity.

### 6.3. Minimally Invasive Techniques

Recent advancements in minimally invasive spine surgery (MISS) have improved the management of facet fractures, reduced surgical morbidity, and allowed faster recovery [37].

(1) Percutaneous pedicle screw fixation: This is ideal for patients with isolated facet fractures and minimizes soft tissue disruption while maintaining stability.
(2) Computer-assisted navigation and robotics: This enhances screw placement accuracy and reduces operative time and is particularly useful for complex subaxial fractures requiring precise screw trajectories.

### 6.4. Comparative Analysis of Treatment Outcomes

Table 4 summarizes a systematic comparison of conservative versus surgical management strategies. It outlines the success rates, complications, and long-term outcomes associated with each approach.

### 6.5. Decision Trees for Management Strategy

We have included decision trees outlining the recommended treatment pathways based on the AO Spine Subaxial Facet Injury Classification to further guide clinical decision-making.

#### 6.5.1. Decision Tree 1: Management of F1 Injury

(1) Conservative treatment: Cervical collar (6–8 weeks) → clinical and radiological monitoring (every 2–4 weeks).
(2) Surgical treatment: Not indicated unless there is evidence of progressive instability, neurological compromise, or persistent pain.

#### 6.5.2. Decision Tree 2: Management of F2 Injury

(1) Conservative treatment: Rigid cervical collar (8–12 weeks) if minimal displacement and stable PLC → clinical and radiological monitoring (every 2–4 weeks) to detect instability.
(2) Surgical treatment: Indicated if progressive symptoms or instability are identified.
  (a) Posterior fixation: Preferred approach using lateral mass screws or pedicle screws depending on fracture severity.
  (b) Anterior reconstruction (ACDF or ACCF): Considered if there is disc herniation or anterior column instability.
  (c) Combined anterior-posterior stabilization: Rarely needed but considered if both anterior and posterior instability are suspected.

#### 6.5.3. Decision Tree 3: Management of F3 Injury

(1) Conservative treatment: Not recommended due to high risk of deformity, neurological compromise, and chronic instability.
(2) Surgical treatment
  (a) Posterior fixation: Primary approach using lateral mass screws or pedicle screws.
  (b) Anterior reconstruction (ACDF or ACCF): Used when significant disc disruption or anterior instability is present.
  (c) Combined anterior-posterior stabilization: Recommended for complex or multi-level instability to provide robust stabilization.

#### 6.5.4. Decision Tree 4: Management of F4 Injury

(1) Conservative treatment: Not recommended due to profound instability and high risk of neurological deterioration.
(2) Surgical treatment:
  (a) Posterior fixation: Preferably using pedicle screws for maximum stability.
  (b) Lateral mass screws: May be used but often requires additional fixation techniques.
  (c) Combined anterior-posterior stabilization: Frequently indicated for severe injuries to achieve adequate stabilization.
  (d) Anterior decompression (ACDF or ACCF) followed by posterior fixation: Recommended when there is extensive vertebral body involvement or traumatic disc herniation.

### 6.6. Expected Recovery Trajectories and Functional Outcomes

Understanding expected recovery trajectories and assessing functional outcomes is essential for optimizing subaxial facet and lateral mass fracture treatment strategies.

#### 6.6.1. Recovery Trajectories

(1) Conservative management: Healing times for minimally displaced F1 fractures are generally favorable, with immobilization for 6–8 weeks followed by a gradual return to normal activities. For F2 injuries managed conservatively, extended immobilization (8–12 weeks) may be necessary, with clinical and radiographic monitoring to detect delayed instability. Potential complications include residual pain, deformity, or progression to instability, especially in cases with incomplete ligamentous healing.
(2) Surgical management: Surgical stabilization is typically followed by a structured rehabilitation program to restore neck mobility and strength. Recovery trajectories depend on the severity of the injury and the type of surgical intervention performed (e.g., posterior fixation, anterior reconstruction, or combined approaches). Early surgical intervention in unstable fractures (F3, F4) is associated with improved alignment, reduced pain, and enhanced functional recovery.

#### 6.6.2. Patient-Reported Functional Outcomes

(1) Functional outcome measures: Commonly used patient-reported outcome measures (PROMs) include the Neck Disability Index (NDI), Visual Analog Scale (VAS) for pain, and SF-36 for quality of life assessment. Surgical management is generally associated with better functional outcomes in unstable fractures, whereas conservative management can yield satisfactory results in stable injuries.
(2) Comparison of conservative vs. surgical management: Studies comparing conservative and surgical management have reported superior functional outcomes following surgical stabilization in F3 and F4 fractures. However, for stable fractures (F1, stable F2), conservative management often results in comparable outcomes without the risks associated with surgery.

### 6.7. Ranking of Treatment Strategies Based on Evidence Levels

We have ranked the available treatment strategies based on their evidence levels to provide more explicit guidance on optimal management approaches. Studies were classified as high level (prospective cohort studies, randomized controlled trials) or low level (retrospective case series, expert opinions).

#### 6.7.1. High-Level Evidence (RCTs, Cohort Studies)

Surgical stabilization for F3 and F4 fractures has demonstrated superior outcomes in terms of stability, pain reduction, and prevention of neurological deterioration. Early intervention with posterior fixation or combined anterior-posterior stabilization is generally recommended for unstable injuries.

#### 6.7.2. Moderate to Low-Level Evidence (Retrospective Studies, Expert Opinion)

Conservative management is commonly employed for F1 and stable F2 fractures. The success of conservative management is highly dependent on fracture morphology, ligamentous integrity, and radiographic stability. Treatment decisions often rely on clinical judgment rather than robust evidence for borderline cases (e.g., F2 injuries with partial ligamentous disruption).

### 6.8. Economic Considerations of Conservative vs. Surgical Management

Understanding the economic implications of different management strategies is essential for making informed clinical decisions.

#### 6.8.1. Conservative Management

Initial treatment costs are generally lower due to noninvasive methods such as cervical collars or halo vests. However, long-term costs may accumulate if complications arise, such as delayed instability, persistent pain, or the need for subsequent surgical intervention. Prolonged immobilization can also result in indirect costs related to lost productivity and extended rehabilitation.

#### 6.8.2. Surgical Management

Surgical treatment involves higher initial costs due to operating room expenses, implants, and hospital stays. However, early surgical intervention for unstable injuries (e.g., F3, F4) may provide better long-term functional outcomes, reducing the need for prolonged rehabilitation or revision surgery. Successful stabilization may also enable an earlier return to work or normal activities, offsetting initial expenses.

#### 6.8.3. Future Considerations

Comparative cost-effectiveness studies are needed to determine the optimal approach for different fracture types, particularly borderline cases (e.g., unstable F2 fractures). As healthcare systems increasingly emphasize value-based care, understanding the economic implications of treatment decisions will become increasingly important.

## 7. Vertebral Artery Injury in Subaxial Facet Injuries (C3–C7)

Vertebral artery injury (VAI) is a critical concern in subaxial cervical spine trauma, particularly in facet dislocations, fractures, or subluxations. The vertebral artery traverses the cervical spine uniquely, making it vulnerable to direct and indirect C3–C7 facet injuries.

### 7.1. Anatomical Considerations of the Vertebral Artery (C3–C7)

The vertebral artery originates from the subclavian artery. It is divided into four segments:

(1) V1 (pre-foraminal segment): From its origin to the transverse foramen of C6.
(2) V2 (foraminal segment): Ascends through the transverse foramina of C6 to C2.
(3) V3 (extracranial segment): From C2 to the dura mater at the foramen magnum.
(4) V4 (intracranial segment): From dural penetration to its termination at the basilar artery.

In the subaxial cervical spine (C3–C7), the vertebral artery mainly courses through the V2 segment as it passes through the transverse foramina. However, its path is not entirely straight—it follows a slight lateral and posterior trajectory, making it susceptible to traction, compression, and laceration in cervical trauma.

### 7.2. Mechanisms of VAI in Subaxial Facet Injuries

VAI in subaxial facet trauma occurs through several mechanisms [9,10,11,12]:

#### 7.2.1. Direct Compression or Stretching

**Figure 8:**
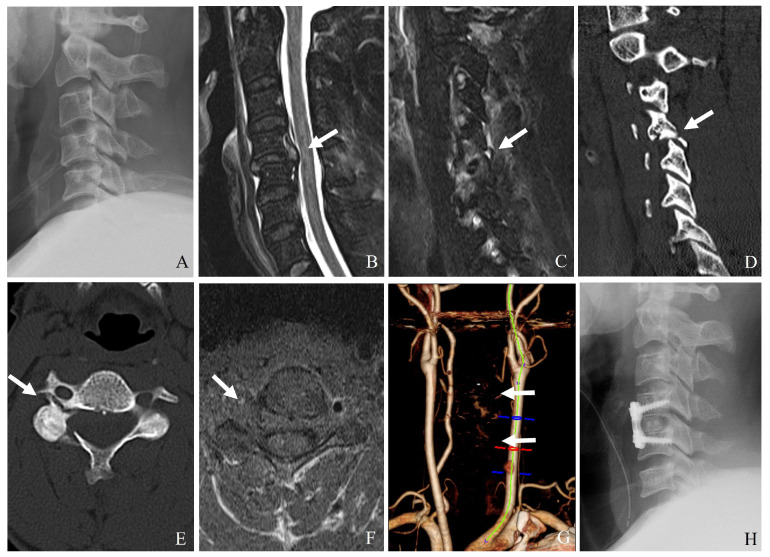
Plain X-ray (**A**), magnetic resonance imaging (**B**,**C**), and computed tomography (**D**,**E**) indicate unilateral facet subluxation, traumatic disc herniation, and fractures of the right facet, pedicle, and lamina at C4–5 (white arrows). Axial magnetic resonance imaging (**F**) and neck computed tomography angiography (**G**) reveal left vertebral artery injury (white arrows). The patient underwent combined anterior cervical discectomy, fusion, and posterior fixation of C4–5 (**H**).

(1) Unilateral or bilateral facet dislocation can cause excessive rotational and translational forces on the vertebral artery, particularly at C3–C6 levels.
(2) Perched or locked facets can lead to excessive stretch or kinking of the artery within the transverse foramen.
(3) Fracture of the lateral mass or pedicle can lead to direct bony impingement on the vertebral artery (Figure 8).

#### 7.2.2. Occlusion from Displacement or Thrombosis

Posterior displacement of the inferior articular process can cause mechanical occlusion. Intimal tears from excessive stretching can result in thrombosis, leading to embolic complications or posterior circulation strokes.

#### 7.2.3. Laceration or Dissection

(1) A facet fracture extending into the transverse foramen may directly lacerate the vertebral artery.
(2) High-energy trauma can cause intimal tears, leading to dissection and secondary stenosis or occlusion.

#### 7.2.4. Hyperextension and Rotational Injury

Forced hyperextension can compress the vertebral artery against the posterior aspect of the vertebral body. Rotational forces can lead to excessive arterial elongation, increasing the risk of intimal damage and thrombus formation.

#### 7.2.5. Penetrating Trauma or Iatrogenic Injury

Examples of direct trauma from penetrating injuries include gunshot wounds and stab wounds. Excessive traction or instrument manipulation can damage the vertebral artery during the open reduction of facet dislocations.

### 7.3. Clinical Implications and Diagnosis

#### 7.3.1. Symptoms: Patients with Vertebral Artery Injury May Present with

(1) Posterior circulation ischemic symptoms (dizziness, vertigo, ataxia).
(2) Stroke-like symptoms (contralateral weakness, dysphagia).
(3) Neck pain with or without neurological deficits.
(4) Delayed-onset symptoms due to progressive thrombosis.

#### 7.3.2. Imaging [13,14,15]

**Figure 9:**
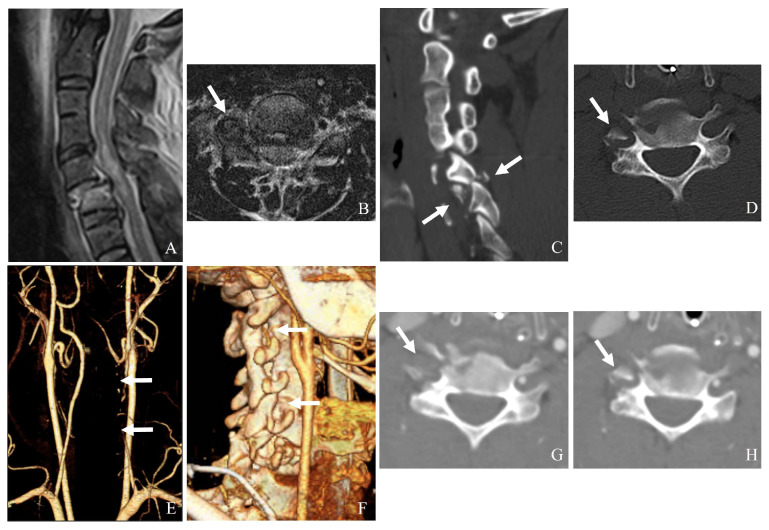
Magnetic resonance imaging (**A**,**B**) and computed tomography (**C**,**D**) demonstrate C5–C6 bilateral facet dislocation, traumatic disc herniation, and a right transforaminal fracture fragment (white arrows). Neck computed tomography angiography (**E**–**H**) reveals a right vertebral artery injury caused by the transforaminal fracture fragment (white arrows).

(1) CT angiography: The gold standard for detecting vertebral artery injuries (Figure 9).
(2) Magnetic resonance angiography: Useful for assessing arterial dissection and thrombus formation.
(3) Digital subtraction angiography: The most sensitive but invasive method.

### 7.4. Management Strategies for VAI [12,16]

**Figure 10:**
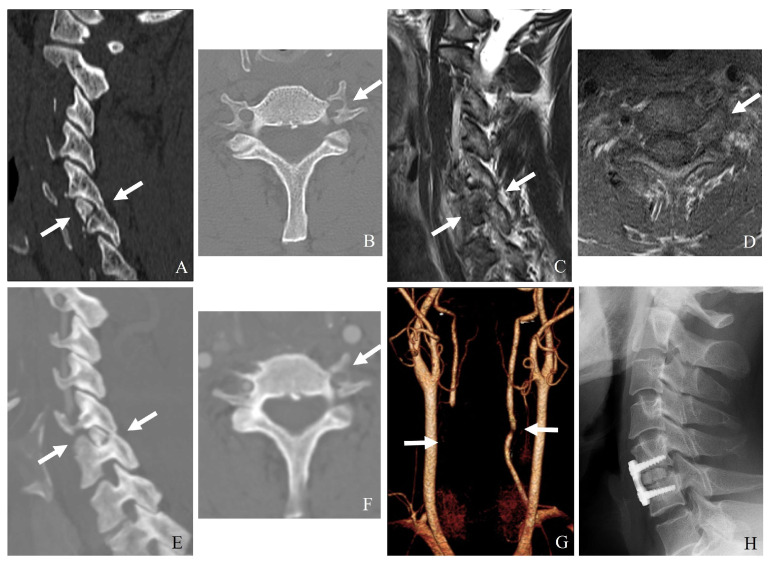
Computed tomography (**A**,**B**) and magnetic resonance imaging (**C**,**D**) show C5–6 unilateral facet dislocation and a left transforaminal fracture fragment (white arrows). Neck computed tomography angiography (**E**–**H**) indicates left vertebral artery injury due to the transforaminal fracture fragment and mild right vertebral artery occlusion (white arrows). The patient underwent anterior cervical discectomy and fusion of C5–C6 (**H**).

Management of VAI includes conservative treatment, surgical considerations, and endovascular interventions (Table 5). Conservative management involves anticoagulation or antiplatelet therapy for minor dissection or thrombosis without neurological deficits, along with regular radiological follow-up. Surgical management requires careful dissection around the transverse foramen during open reduction of facet dislocations and caution when fractures involve the transverse foramen to prevent further injury (Figure 10). Endovascular interventions, such as stenting for vessel narrowing or dissection and coil embolization for pseudoaneurysms or active bleeding, may be necessary for severe arterial damage.

### 7.5. Prevention of Stroke in VAI

#### 7.5.1. Short-Term Stroke Prevention

(1) Immediate initiation of antiplatelet therapy (aspirin or clopidogrel) for grade I–II injuries.
(2) Avoid abrupt cervical spinal manipulation before confirming vertebral artery patency.
(3) Depending on the risk of bleeding, heparin or warfarin may be considered in patients with significant stenosis or thrombus formation.

#### 7.5.2. Long-Term Stroke Prevention

(1) Continued antiplatelet therapy for 3–6 months, followed by re-evaluation with CTA or MRA.
(2) Endovascular stenting for persistent pseudoaneurysm or progressive stenosis.
(3) Lifestyle modifications (blood pressure control, smoking cessation, cholesterol management).
(4) Rehabilitation and physical therapy to prevent secondary falls and injuries.

### 7.6. Complications and Prognosis

(1) Ischemic stroke is the most severe complication, occurring in 7–24% of patients with untreated or poorly managed VAI.
(2) Embolic events can lead to posterior circulation infarcts, particularly affecting the brainstem, thalamus, and cerebellum.
(3) Chronic vertebrobasilar insufficiency can result from vascular remodeling and progressive stenosis.

## 8. Future Directions and Defining Instability

### Instability Definition Challenges

Despite the widespread use of classification systems such as the AO Spine Subaxial Facet Injury Classification, spinal instability remains a somewhat subjective and poorly defined parameter, particularly for intermediate injuries like F2 fractures. While classifications often attempt to categorize instability based on structural findings, such as displacement or ligamentous injury, they do not provide a universally accepted biomechanical threshold clinicians can reliably apply across different patient populations. The absence of consistent criteria for defining instability complicates treatment decision-making, especially when distinguishing between injuries that may benefit from conservative management versus those that require surgical intervention. Studies often rely on indirect measures of instability, including imaging findings, neurological symptoms, or subjective clinical judgment. However, there remains considerable variability in how instability is assessed and reported, which underscores the need for more objective and reproducible criteria.

Additionally, the literature relies heavily on retrospective studies and expert opinion, particularly in managing F2 injuries where ligamentous involvement may not be fully appreciated through static imaging alone. This lack of high-quality evidence limits the ability to establish firm guidelines for clinical practice, resulting in variability in treatment approaches. Addressing these gaps will require a more structured and evidence-based approach. Specifically, future research should prioritize the development of objective criteria for defining instability, possibly through advanced imaging techniques such as dynamic MRI or CT to capture vertebral motion and ligamentous integrity under stress conditions. Prospective multicenter cohort studies and randomized controlled trials comparing conservative and surgical management strategies, particularly for F2 fractures, would provide valuable data to guide clinical decision-making. Additionally, integrating standardized patient-reported outcome measures (PROMs) such as the Neck Disability Index (NDI) and SF-36 into future research would enhance our understanding of functional recovery and patient satisfaction.

## 9. Conclusions

Subaxial minimal facet and lateral mass fractures of the subaxial cervical spine require a nuanced approach to diagnosis and treatment. While most cases can be managed conservatively, recognizing subtle instability is crucial to prevent complications. Surgical intervention should be reserved for cases with confirmed instability, progressive deformity, or neurological deficits. Vertebral artery injury in subaxial cervical facet injuries is a serious but often underappreciated complication. Understanding the anatomical course of the vertebral artery and the mechanisms of injury at C3–C7 is essential for early diagnosis and appropriate management. A high index of suspicion, combined with advanced imaging and a multidisciplinary approach, is crucial to prevent catastrophic neurological outcomes. Advancements in imaging, minimally invasive techniques, and biomechanical research continue to shape treatment paradigms.

## Figures and Tables

**Table 1:** Facet joint anatomy and stability.

Aspect	Description
Facet Joint Structure	Synovial articulations between superior and inferior articular processes of adjacent vertebrae
Orientation	Oriented at approximately 45 degrees in the coronal plane
Movements Facilitated	Controlled flexion, extension, and rotational movements prevent excessive anterior translation
Supporting Structures	Fibrous capsule, ligamentum flavum, interspinous ligaments, posterior longitudinal ligament (PLL)
Stability Importance	The integrity of the facet joint is critical for maintaining the segmental motion of the cervical spine

**Table 2:** Disc and ligamentous complex in stability.

Aspect	Description
Intervertebral Disc	Located between vertebral bodies, it contributes to the stability and load distribution.
Anterior Column	Includes anterior longitudinal ligament (ALL) and vertebral bodies with intervertebral discs.
Middle Column	Includes posterior longitudinal ligament (PLL) and posterior aspect of vertebral bodies.
Posterior Column	Includes facet joints, ligamentum flavum, interspinous, and supraspinous ligaments.
Functions of Ligaments	- ALL prevents excessive extension. - PLL prevents excessive flexion. - Ligamentum flavum and interspinous ligaments provide posterior tension and prevent excessive separation.

**Table 3:** AO Spine Subaxial Facet Injury Classification (C3–C7).

Classification	Definition	Radiologic Findings	Clinical Significance
F1 Fracture	Nondisplaced or minimally displaced facet fracture	CT: Hairline or incomplete fracture without subluxation or misalignment. MRI: Minimal or no disruption of the DLC.	Mechanically stable; treated conservatively with immobilization.
F2 Fracture	Displaced facet fracture without significant instability	CT: Displaced fracture, but no complete subluxation or dislocation. Fracture extends < 40% into vertebral body. MRI: Partial PLC injury, intact DLC.	May be treated conservatively; surgical stabilization if symptoms progress.
F3 Fracture	Unstable facet fracture with significant displacement or dislocation	CT: Completely displaced or perched facet with significant translation and rotation. Fracture extends ≥ 40% into vertebral body. MRI: Complete disruption of PLC and DLC.	Highly unstable; surgical intervention usually required to prevent neurological deterioration.
F4 Fracture	Comminuted facet fracture with severe instability	CT: Highly comminuted fragment with vertebral displacement. MRI: Complete ligamentous disruption and extensive soft tissue injury.	Requires immediate surgical intervention; posterior fusion or combined stabilization.

**Table 4:** Comparison of conservative vs. surgical management.

Parameter	Conservative Management (F1, F2)	Surgical Management (F2, F3, F4)
Conservative Management	High in F1; moderate in F2 (if stable)	High in F3, F4; moderate in F2
Complications	Delayed instability, deformity, kyphosis	Surgical site infection, hardware failure
Long-term Outcomes	Favorable in stable fractures (F1)	Favorable in appropriate stabilization
Risk of Neurologic Deficit	Low (F1); moderate (F2 if instability exists)	High (F3, F4) without intervention
Indications	Stable fractures (F1, stable F2)	Unstable fractures (F2, F3, F4)

**Table 5:** Management strategies for vertebral artery injury.

Management Type	Indications	Treatment
Conservative Management	Minor dissection or thrombosis without neurologic deficits	Anticoagulant or antiplatelet therapy (e.g., aspirin, heparin) Close radiological follow-up
Surgical Considerations	Open reduction of facet dislocations Transverse foramen involvement in fractures Severe arterial disruption	Careful; dissection around the transverse foramen Avoid exacerbating vertebral artery injury Endovascular interventions (e.g., stenting, embolization)
Endovascular Interventions	Vessel narrowing or dissection Pseudoaneurysm or Active bleeding	Stenting for vessel stabilization Coil embolization

## Data Availability

Not applicable.

## Abbreviations

The following abbreviations are used in this manuscript:

CT	Computed tomography
MRI	Magnetic resonance Imaging
ALL	Anterior longitudinal ligament
PLL	Posterior longitudinal ligament
DLC	Disco-ligamentous complex
PLC	Posterior ligamentous complex
ACDF	Anterior cervical discectomy and fusion
ACCF	Anterior cervical corpectomy and fusion
MISS	Minimally invasive spine surgery
CTA	Computed tomography angiography
VAI	Vertebral artery injury
